# When STING Meets Viruses: Sensing, Trafficking and Response

**DOI:** 10.3389/fimmu.2020.02064

**Published:** 2020-09-29

**Authors:** Zhaohe Li, Siqi Cai, Yutong Sun, Li Li, Siyuan Ding, Xin Wang

**Affiliations:** ^1^Key Laboratory of Marine Drugs of Ministry of Education, School of Medicine and Pharmacy, Ocean University of China, Qingdao, China; ^2^Center for Innovation Marine Drug Screening and Evaluation, Pilot National Laboratory for Marine Science and Technology, Qingdao, China; ^3^Marine Biomedical Research Institute of Qingdao, Qingdao, China; ^4^Department of Molecular Microbiology, School of Medicine, Washington University in St. Louis, St. Louis, MO, United States

**Keywords:** STING, DNA viruses, cellular trafficking, post translational modifications, immune responses

## Abstract

To effectively defend against microbial pathogens, the host cells mount antiviral innate immune responses by producing interferons (IFNs), and hundreds of IFN-stimulated genes (ISGs). Upon recognition of cytoplasmic viral or bacterial DNAs and abnormal endogenous DNAs, the DNA sensor cGAS synthesizes 2’,3’-cGAMP that induces STING (stimulator of interferon genes) undergoing conformational changes, cellular trafficking, and the activation of downstream factors. Therefore, STING plays a pivotal role in preventing microbial pathogen infection by sensing DNAs during pathogen invasion. This review is dedicated to the recent advances in the dynamic regulations of STING activation, intracellular trafficking, and post-translational modifications (PTMs) by the host and microbial proteins.

## Introduction

The immune response is a complicated process in which the body defends against pathogen infections and confines the disease progression, leading to the eventual recovery, and conferring protective immunity. Innate immunity is the first line to resist viral invasion. A myriad of host factors, such as interferons (IFNs), cytokines, and chemokines, respond quickly to viral invading, and trigger adaptive immunity (1). Due to the special biological features of viruses and their unique relationships with host cells, antiviral immunity not only shares commonalities with antibacterial immunity but also has unique characteristics. Invading viruses trigger innate immunity during and after entry into host cells via germline-encoded molecules termed pattern recognition receptors (PRRs), which detect pathogens by recognition of their conserved molecular structures, called pathogen-associated molecular patterns (PAMPs) (2). In this process, different PRRs jointly participate in the complicated and delicate immune responses by collaboration between multiple PRRs and their downstream factors. Until now, stimulator of interferon genes (STING) is the most important adaptor protein in immune responses against DNA viruses, in cooperation with other well-identified molecules, including cGAS, TBK1, IRF3, and NF-κB (3–5).

## DNA Virus Infection, Immune Response, and Diseases

To date, more than 6,000 types of viruses have been identified according to the International Committee on Taxonomy of Viruses (ICTV). There are approximately 400 kinds of viruses of human health concerns and many are double-stranded DNA (dsDNA) viruses or retroviruses. For instance, the retrovirus human immunodeficient virus (HIV) is considered as a DNA virus here because of the viral dsDNA produced by reverse transcription process, leading to Acquired Immune Deficiency Syndrome (AIDS). Similarly, chronic infection with human hepatitis B virus (HBV), which is harboring a partial double-stranded genomic DNA and belongs to *Hepadnaviridae*, leads to liver fibrosis and cancers (6). Other pathogenic human DNA viruses mostly belong to the *Poxviridae*, *Herpesviridae*, *Adenoviridae*, *Papillomaviridae*, and *Polyomaviridae* families. In the *Herpesviridae* family, human cytomegalovirus (HCMV) causes immunocompromised symptoms of the brain, liver, spleen, and lung (7). Infection by the herpes simplex virus 1 (HSV-1) results in painful blisters or ulcers (8). What’s more, it might lead to more serious symptoms including encephalitis. HSV-2 infection is a typical sexually transmitted disease (STD) with the symptom of different genital warts (9). Epstein-Barr virus (EBV) is involved in numerous types of lymphomas and gastric cancers (10). Kaposi’s sarcoma–associated herpesvirus (KSHV) is found in Kaposi’s sarcoma, primary effusion lymphoma, and multicentric Castleman’s disease. The incidence of Kaposi’s sarcoma is much higher in immunosuppressed individuals, because of the deficiency of host immunity (10, 11). Especially, Kaposi’s sarcoma has a high fatality rate among AIDS patients (12). Virulent adenoviruses lead to the common cold, fever, sore throat, acute bronchitis, pneumonia, and neurologic disease (in rare cases) (13–15, 187). In the *Papillomaviridae* and *Polyomaviridae* families, high-risk human papillomaviruses (HPVs) are admittedly oncogenic and significantly related to cervical cancer and head and neck cancers (16), while low-risk HPVs are responsible for anogenital condyloma, genital warts, and other skin diseases (17). Merkle cell polyomavirus (MCPyV) integration is found in Merkle cell carcinoma. JC polyomavirus and BK polyomavirus are found in organ transplant patients (18, 19). Therefore, a thorough understanding of the arms race between DNA viruses and host immunity is required to develop therapeutic strategies for viral infections.

Innate immunity is vital to restrict viral infections at the early stages of host antiviral immunity (20). After the invasion, viral PAMPs stimulate IFN production in a variety of cells, which possess a broad-spectrum antiviral effect (21). Thus, they would induce antiviral albumin to block viral propagation (22). For innate immune responses to viral invasion, although PRRs and IFN signaling are constituently components in nearly all somatic cells to control early infections in our body, it is believed that leukocytes are the protagonists in the stage to clear propagating viruses, by either secreted IFNs, and cytokines or cell killing. In innate immune cells, macrophages are tissue-residents, and clear virions and infected cells by phagocytosis. Natural killer cells (NK cells) account for 5–10% of the total number of lymphocytes and are constantly undertaking “patrol” tasks in the body. Infected host cells that lack MHC-I molecules are within the scope of NK cell attacking (23, 24). Additionally, dendritic cells (DCs) are the main bridge between innate and adaptive immunity by antigen presentation. DCs are also the major producers of IFNs in the peripheral blood (25, 26).

## Cellular Sensors of Abnormal DNA

Several DNA sensing PRRs have been characterized so far, including Toll-like receptors (TLRs), NOD-like receptors (NLRs), C-type lectin receptors (CLRs), and cytosolic DNA sensors including cyclic GMP–AMP synthase (cGAS), IFN-γ (IFN-γ)-inducible protein 16 (IFI16), heterogeneous nuclear ribonucleoprotein A2/B1 (hnRNPA2B1), absent in melanoma 2 (AIM2), DNA-dependent activator of IRFs (DAI), RNA polymerase III, DEAD box helicase 41 (DDX41), DEAH box protein 9 (DHX9)/DEAH box protein 36 (DHX36), leucine-rich repeat flightless-interacting protein 1 (LRRFIP1), Ku70, and Sox2 (3, 27, 28). As the first discovered DNA recognition molecule, DAI binds to dsDNA and induces type I IFN (IFN-I) (29, 30). However, the knockdown of DAI does not affect the innate immune response of mice to B-DNA stimulation in later studies, raising controversy (31). AIM2 is an IFN induced cytosolic protein containing a pyrin domain (PYD) and a HIN200 domain. The HIN domain promotes its binding to DNA. The PYD binds to ASC, the apoptosis-associated speck-like protein containing a caspase recruitment domain (CARD), forming an activated caspase-1 inflammasome to promote releases of IL-1β, and IL-18 (32). RNA polymerase III converts dsDNA poly (dA:dT) into 5′- triphosphate double-stranded RNA, delivering signals to the RIG-I pathway (33, 34). DDX41 (DEAD box polypeptide 41) promotes IFN and IFN-stimulated genes (ISGs) expression in a STING-dependent way. It recognizes intracellular DNA or bacterial c-*di*-GMP and c-*di*-AMP and then activates IRF3 by TBK1 (35, 36). IFI16 is predominantly a nuclear protein sensing abnormal DNA in the nucleus (37). HnRNPA2B1 is another recently reported nuclear initiation factor that detects and limits DNA virus infection (38).

Potentiating signals from many other DNA sensors and cyclic dinucleotides (CDNs) binding, places STING a nodal position to restrict DNA viruses. The first promised DNA sensor IFI16 implicating in IFN induction by DNA stimulation localizes both in the nucleus and the cytoplasm but may sense abnormal DNA in the nucleus (39), because HSV-1 ICP0 re-localizes IFI16 from the nucleus to the cytoplasm, hampering IFN responses to viruses (40). Active IFI16 recruits STING to facilitate a TBK1-dependent gene induction. Knockdown of IFI16 or its mouse ortholog p204 impairs IFN-I induction in response to dsDNA or HSV-1 genomic DNA (39). As the PYHIN protein AIM2, IFI16 can activate inflammasome-mediated immune responses (41, 42). DDX41 scaffolds DNA and STING in the cytosol for ISG induction. Knockdown of DDX41 blocks TBK1 phosphorylation, and IRF3- or NF-κB- dependent gene expression in mouse DCs (35). Nuclear protein Ku70 and hnRNPA2B1 also induce IFN expression by STING (43, 44). Ku70 is an important component in the DNA damage repair (DDR) machinery (45), collaborating with STING to maintain the host genomic integrity and clear damaged cells. However, DNA viruses utilize host DDR components during viral DNA replication (46, 47), and the crosstalk between Ku70-STING might also contribute to antiviral immune responses. The newly-identified DNA sensor hnRNPA2B1 senses viral genomic DNA in the nucleus. Undergoing homodimerization and demethylation at the Arg226 site by JMJD6, hnRNPA2B1 translocate into the cytoplasm, where STING and TBK1 are recruiting. HnRNPA2B1 simultaneously promotes cGAS, IFI16, and STING expression, which in turn amplifies cGAS-STING signaling (38). It is noting that STING signals may crosstalk with the RIG-I-MAVS pathway during viral infections (48, 49).

The discovery of cGAS in DNA sensing process greatly expanded the understanding of intracellular exogenous or abnormal DNA sensing (3). Unlike other DNA sensors, cGAS catalyzes and releases the second messenger cGAMP from ATP and GTP after DNA recognitions, instead of directly binding to the adaptor protein STING (50, 51). Cytosolic cGAMP inserts into STING dimer and induces a conformation change, leading to the exposure of C-terminal tail (CTT) of STING for TBK1 recruitment (52, 53). Moreover, cGAS-deficient mice show a complete loss of IFN production in response to DNA stimulation or DNA virus infection (HSV-1, vaccinia virus, and murine γ herpesvirus). It indicates the importance of cGAS in DNA-induced immune responses. cGAMP can be transferred from infected cells to uninfected neighboring cells through gap junctions or exosomes, where it amplifies immune responses to DNA stimulation independent on IFN signaling (54). Leucine-rich repeat containing 8 VRAC subunit A (LRRC8) volume-regulated anion channel facilitates this process (55). These findings uncover a novel host strategy that rapidly conveys antiviral immunity to bystander cells independent of the paracrine signaling of IFNs.

RNA viruses, such as dengue virus, induce mitochondria DNA (mtDNA) leakage into the cytosol and trigger STING signaling (56, 57). This interesting observation partially explained the reduction of IFN expression response to RNA virus infection in STING-deficient cells. Adaptor protein TRIF facilitates STING signaling by the interaction with STING on its carboxyl-terminal domains to promote its dimerization and translocation (58). The crosstalk between adaptor protein STING, MAVS, and TRIF become interesting and elucidated now. Noticeably, these adaptor proteins share some common behaviors, such as phosphorylation patterns and oligomerization (59–61). Although these adaptor proteins seem to all play roles in detecting cytosolic DNA, their contributions to DNA-mediated gene induction are either partial or cell type specific.

## The Structure and Subcellular Localization of STING

Abnormal cytosolic DNA molecules trigger a dsDNA sensing process, which consequently induces IFNs and ISGs expression (62). As mentioned above, nuclear DNA sensors also potentiate the signaling in the cytoplasm after intracellular translocation. STING locates in the ER and consists of four transmembrane regions, which is expressing in a variety of endothelial cells, epithelial cells, and hematopoietic hepatocytes (61, 63). Human STING encodes a protein of 379 amino acids (aa), containing a predicted transmembrane portion (1-173aa) in the N-terminus and an intracellular soluble portion (174-379aa) in the C-terminus (64). The N-terminus regulates its cellular localization and homodimerization, since the transmembrane domains cross the ER membrane (61, 65). The C-terminal domain (CTD) functionally docks downstream molecules, including TBK1/IKKε, and IRF3/IRF7 (66–68). To potentiate the signaling, the native ligand cGAMP binds to the V-shaped hydrophilic pocket in STING dimer (50). Undergoing a conformational change, the hidden CTT of STING is exposed to TBK1 and IRF3 (69–73). During this process, STING is transported from the ER to the ER-Golgi intermediate compartment (ERGIC), Golgi, and then perinuclear regions (74). Although the cGAMP induced STING activation via a closed conformation, the artificial agonist diABZI activates STING with an open conformation (75). It is still unclear that if STING conformation changes are required for the following intracellular translocation. Studies should be pursued to elucidate the details.

The classical STING signaling starts on the appearance of DNA in the cytoplasm, which is considered as an abnormal signal. Once triggered by free DNA in cytoplasm, cytoplasmic cGAS catalyzes the synthesis of cGAMP to activate STING (50, 76). Alternatively, other PRRs directly bind to STING, such as hnRNPA2B1 and IFI16 (38, 77). After activation, STING travels to the endosome through the ER and the Golgi apparatus via intracellular trafficking or autophagy process (78). STING dimers are closely arranged side by side in the lipid membrane under the active state. Dimerized STING can be connected to adjacent dimers, and these connections are stabilized by connecting the dimer’s ring at its interface (79). Without cGAMP bound, the connecting element may stabilize the inhibitory direction of the interface loop. It is hypothesized that the rearrangement of the connecting element on cGAMP binding is related to the STING activation. Although it is still hard to understand how to form chemical bonds between adjacent STING dimers for the side by side oligomer maintenance, there is no other better explanation at this moment.

TBK1 dimer associates with STING at the perinuclear region after cellular trafficking. It docks on top of the cGAMP binding domain of STING. This interaction is mediated by the conserved eight amino acid residues in the CTT domain of STING, which is highly flexible, and hard to be crystalized (72). The peptide linker between cGAMP binding pocket and C-terminus of STING, allows TBK1, and STING to adopt different orientations with each other (50). The ligand cGAMP might be an initiator in the pathway and not needed in the following intracellular trafficking and TBK1 binding. Thus, cGAMP binds to dimerized STING in ER, triggers its conformation change and oligomerization to initiate signaling (50, 80). In the process, STING is transported to the ERGIC, Golgi, and perinuclear regions, where it meets downstream factors, including protein kinase TBK1/IKKε, transcription factors IRF3 and NF-κB, and other cellular factors. Ultimately, IRF3 is phosphorylated by TBK1 and enters into the nucleus to induce IFN and cytokine production (4, 81–84).

## STING-Related Signaling Pathway

**FIGURE 1:**
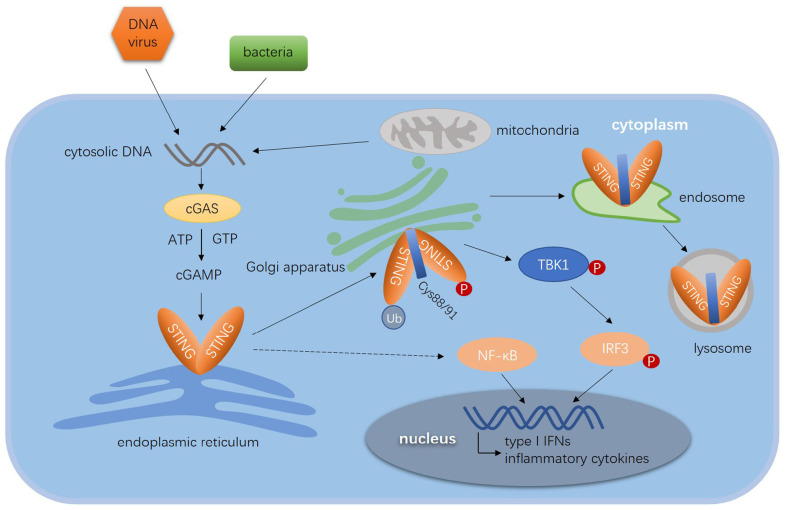
Diagram of STING-mediated immune response to viruses. The cytosolic dsDNA derived from DNA viruses, bacteria CDNs and mitochondria are sensed by cGAS, which catalyzes ATP and GTP to generate cGAMP. Cyclic GAMP directly binds to the pocket of STING dimer and initiates the translocation of STING. STING translocates from the ER to ERGIC, Golgi apparatus and endosome, where it is degraded in the lysosome. The phosphorylation, ubiquitination, and palmitoylation are essential for the activation of STING. The activated STING dimer recruits TBK1 to form the translocation complex. By recruiting and phosphorylating IRF3, the complex promoted IRF3 to entry into nucleus. STING induces the expression of type I IFN genes and other pro-inflammatory cytokines through the TBK1–IRF3 axis and NF-κB signal pathway.

As shown in Figure 1, in the presence of cytosolic DNA, STING translocates sequentially from the ER to ERGIC, Golgi apparatus, and eventually relocates to perinuclear regions where activated STING recruits TBK1 (63, 74). TBK1 phosphorylates and activates IFN regulatory factors (IRFs) and NF-κB, which induces IFN-I, IFN-III, and other pro-inflammatory genes (85, 86). IRF3 and TBK1 dock on polymerized STING complex, thus phosphorylated IRF3 dissociates from the complex and translocate into the nucleus to potentiate gene transcription (51, 84, 87). The regulation of STING signaling mainly focuses on the activation, trafficking, post-translational modifications (PTMs), and downstream pathway. Notably, TOLLIP exerts an important role in STING-mediated immune response and maintain the immune homeostasis. As a stabilizer of STING, TOLLIP interacts with STING directly and maintain the stabilization of STING protein by inhibiting the ER stress sensor IRE1α which suppresses resting-state of STING turnover. Knockdown of TOLLIP reduced the phosphorylation of IRF3 (88). In addition to the activation of STING signaling pathway, STING-mediated immune response also needs to maintain the stabilization of STING protein to ensure an effective response.

As activation, silencing is also critical in signal transduction. The negative feedback loop of STING signals is not clearly understood. To prevent chronic signaling, the active STING together with TBK1 are eventually degraded in a lyso-endosome dependent way (53, 84). STING colocalizes with Rab7 containing vesicles, which are late endosomes or lysosomes, but not early endosomes (Rab5-containing vesicles), or recycling endosomes (Res; Rab11-containing vesicles). Inhibiting acidification of the endo-lysosome pathway prevents activation-induced STING degradation (89, 90). Moreover, cytosolic dsDNA would be cleared in STING induced autophagosomes to prevent chronic cGAS activation. Cells deficient in either cGAS or STING fail to induce autophagy in response to dsDNA (91, 92). In macrophages, the autophagosomal marker LC3 colocalizes with dsDNA as well as cGAS, STING, and TBK1, suggesting the direct role of autophagy in dsDNA clearance and STING degradation (93–95). Cyclic GMP-AMP would be degraded by the extracellular phosphodiesterase ENPP1 to terminate STING signals (96). Besides, it is reported that the cytosolic nuclease poxins (poxvirus immune nucleases) from the vaccinia virus and its homologs from moths, butterflies, and their pathogenic baculovirus, also act as cGAMP-degrading enzymes to prevent cGAS-STING activation (97). More detailed regulation by ubiquitination-mediated degradation would be discussed later. The STING signaling is negatively regulated by protein degradation as well as clearance of the stimulus. Besides ubiquitination, many other posttranslational modifications regulate STING signal transduction and the crosstalk of the STING pathway with other cellular processes. Understanding these mechanistic details may be important for uncovering STING intracellular trafficking and signal transduction.

## STING Trafficking and ISG Induction

**FIGURE 2:**
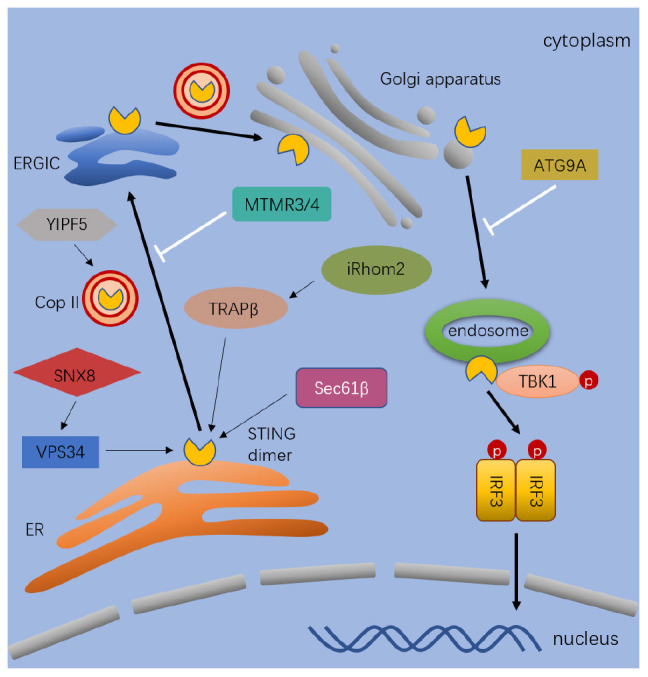
The process and regulation of STING trafficking. After stimulated by cytosolic dsDNA, STING dimer exist from the ER to ERGIC, Golgi, and endosomes. The process of trafficking is mediated by diverse proteins. The thick black arrows indicate the pathway that lead to activation and trafficking of STING. The thin black arrows indicate the regulators which positively regulates the trafficking of STING. The white arrows indicate the regulators which negatively regulates the trafficking of STING. Full name of the abbreviations: VPS34 (Vacuolar protein sorting-associated protein 34); SNX8 (Sorting nexin-8); YIPF5 (Yip1 Domain Family Member 5); MTMR3/4 (Myotubularin Related Protein 3); TRAPβ (Translocon-associated protein β); Sec61β (SEC61 Translocon Subunit Beta); iRhom2 (inactive rhomboid 2); ATG9A (Autophagy-related protein 9A); and Cop II (Coat protein II).

STING trafficking is critical in IRF3 and NF-κB induced ISG expression in response to cytosolic DNA (98). As shown in Figure 2, it is regulated by multiple factors and has not been fully elucidated yet. STING mostly locates in the ER and partially in the mitochondria and mitochondria-associated membranes in resting cells (48, 63, 99). Immediately after ligand binding, dimeric STING translocates between intracellular membranes, from the ER to ERGIC, Golgi apparatus, and perinuclear microsomes or punctate structures (74, 100, 101). Constitutively active STING mutants aggregate in the ERGIC in the absence of ligands, suggesting the ligand-binding itself is not required during the intracellular trafficking process (100). To date, many proteins are known to involved in the regulation of STING trafficking, including the translocon-associated protein β (TRAPβ), the translocon adapter Sec61β, exocyst complex component Sec5, iRhom2, SCAP, SNX8, and YIF5 (63, 102–106). Because STING mutant induced disordered STING translocation and ligand-independent activation contributes to autoinflammatory and autoimmune diseases in patients (107, 108), detailed investigations of STING trafficking become both biological and clinical meaningful (100).

Several pathogen-encoded antagonists of STING have been characterized. The ERGIC localizes between the ER exit sites and the Golgi apparatus as a bridge. The ERGIC sorts ER-derived COPII vesicles for anterograde transport to the Golgi or retrograde transport to the ER (109). The *Shigella* effector protein IpaJ efficiently inhibits gene induction by blocking STING trafficking from the ER to the ERGIC via de-myristoylating the ARF1 GTPase. After exiting from the ERGIC or Golgi, STING translocates to perinuclear punctate structures where it meets TBK1. The VirA protein from *Shigella* blocks STING trafficking from the ERGIC to Golgi by hydrolyzing the Rab1-GTP to Rab1-GDP (74). Even in the presence of STING ligands, inhibition of the translocation either from the ER to ERGIC by IpaJ, or from the ERGIC to Golgi by VirA, hampers STING induced IFN-I expression (99, 100).

Studies on iRhom2 furtherly elucidate the intracellular trafficking in STING signal transduction. iRhom2 is originally reported to promote the trafficking of TACE (TNFα convertase) from the ER to the cell surface and facilitates LPS induced TNFα expression (110). Recent studies are showing that iRhom2 is essential to the immune responses to DNA viruses (106), which is transported from the ER to ERGIC/Golgi apparatus and perinuclear punctate structures together with STING after HSV-1 infection. STING fails to leave ER in iRhom2 deficiency cells, suggesting that viral DNA induced STING translocation is dependent on iRhom2. iRhom2 might adapt STING to interact with TRAPβ, an important translocon-associated protein because knockdown of TRAPβ inhibits STING trafficking and gene induction (106).

In addition to proteins, STING trafficking is also regulated by small molecules, such as phospholipids. Cellular levels of PtdIns (phosphatidylinositol) and PtdIns5P are regulated by myotubularin related protein MTMR3 and MTMR4, which dephosphorylate 3′ position in phosphatidylinositol (PtdIns). MTMR3 and MTMR4 generate PtdIns5P and PtdIns from PtdIns (3, 5) and PtdIns3P, respectively, (111). Increased PtdIns3P is accumulated in enlarged cytosolic puncta in MTMR3 and MTMR4 double knockout (DKO) cells, and STING is aberrantly accumulated in PtdIns3P positive puncta after DNA stimulation. In DKO cells, STING rapidly translocates from the ER to Golgi and produces an enhanced IFN expression in response to IFN-stimulatory DNA (ISD) and HSV-1 infection (111). As shown in Figure 2, MTMR3 and MTMR4 are suppressing STING trafficking in response to DNA stimulation by regulating cellular phospholipid metabolism.

STING is tracking the native vesicular transporters in response to the stimulus, which might inherit from its ancestral functions in autophagy machinery (112). YIPF5 maintains the integrity of Golgi and ER (113, 114), and low levels of YIPF5 are still able to preserve a relatively normal ER network (115). It is essential for viral or intracellular DNA triggered production of IFN and ISGs, by interacting with both STING and components of COPII to facilitate STING recruitment into COPII-coated vesicles and the cellular trafficking from the ER to the Golgi apparatus (105). SNX8, a protein involving in endocytosis and endosomal sorting, belongs to the sorting nexin protein family, which is previously found as a component of IFNγ-triggered non-canonical signaling pathway (116, 117). SNX8 boosts DNA-triggered innate immune responses by recruitment of class III phosphatylinositol 3-kinase VPS34 to STING (118). VPS34 is the key component in STING trafficking from the ER to perinuclear microsomes. SNX8^–/–^ mice fail to respond to HSV-1 infection and exhibiting a lower level of serum cytokines and higher viral titers in mouse brains (118). NLRC3 (nucleotide-binding, leucine-rich-repeat containing protein) negatively regulates STING translocation in response to DNA viral infection. In the presence of NLRC3, STING trafficking to the perinuclear region is prevented (119). NLRC3 is originally reported as an inhibitor in the PI3K-mTOR pathway (120). It is suggesting that STING trafficking may share some common factors in mTOR signals.

In addition to STING, TBK1 and IRF3 also translocate to perinuclear regions in dsDNA stimulated cells. Since the integration of TBK1, IKKs, IRF3, and NF-κB is a later event in the evolutional history of the STING pathway, it is hypothesized that the translocation of TBK1 and IRF3 is dependent on STING trafficking (112). In perinuclear puncta, STING recruits TBK1 to activate transcription factor IRF3 by phosphorylation. STING deficiency leads to the retention of TBK1 to perinuclear regions after dsDNA stimulation. The ATPase inhibitor Brefeldin A (BFA) prevents STING-mediated IRF3 phosphorylation and ISG expression by restriction of STING trafficking (121). Sec5, the exocyst complex component, is essential for the antiviral responses to recruit and activate TBK1 (122). DNA stimulation leads to translocation of STING to Sec5-containing endosome from the ER or ERGIC (63, 102). During this process, TRAPβ, and Sec61β are needed. TMED2 that belongs to the transmembrane emp24 domain/p24 (TMED) family promotes STING-TRAPβ interaction and enhances STING trafficking and gene induction (123). Knockdown of TRAPβ, Sec61β, and Sec5 inhibits STING dependent gene expression. These studies suggest that STING links cytosol DNA stimulation to TBK1 activation through the intracellular trafficking between the ER and perinuclear punctate structures. Similarly, SCAP recruits IRF3 into STING complex and translocates from the ER to perinuclear microsomes after viral infection (124). It could be interesting to disrupt scaffold proteins between STING-IRF3 and STING-TBK1 to figure out the driver factor in the orchestrated process.

Notably, endocytosed cyclic di-nucleotides (eCDNs) released from damaged or dying infected cells could activate bystander cells. Upon binding to eCDNs, cGAS undergoes a conformational change and promotes its interaction with STING. It facilitates the formation of eCDNs/cGAS/STING perinuclear signalosomes to enable STING activation (125). This finding provides an insight into the differences between eCDNs and cGAMP in STING activation and trafficking. Detailed molecular mechanisms are still remaining to be elucidated.

## Viral Infection and STING Trafficking

Viral DNA and virus-induced leakage of mtDNA trigger STING activation and trafficking (98, 126). Viruses have to evolve certain strategies to defeat host immunity for efficient infection. For example, HSV-1 encodes series of proteins to antagonize STING signals, including viral ubiquitin ligase ICP0, deubiquitylase (DUB) UL36USP, protein kinases (US3, VP24), and protein-protein interaction inhibitors (PPis) (127). Since ubiquitination regulates protein trafficking, it is natural to wonder if viral ICP0 and UL36USP would change intracellular trafficking of STING and components in STING signaling. Adenovirus E1A and human papillomavirus E7 inhibit cGAS-STING signals by direct interaction between the LXCXE motifs of viral oncoproteins and STING (128). NS4B of hepatitis C virus and NS2B3 protein of dengue virus directly cleave STING (129, 130). VP24 of HSV-1 and vIRF1 of KSHV impair STING-TBK1 interaction (128, 131). HSV-1 VP1-2 and HTLV-1 Tax protein deubiquitinate STING and inhibit its downstream signals (132, 133). Interestingly, the conserved hemagglutinin fusion peptide of RNA virus influenza virus A (IVA) interacts STING and abolishes STING dependent IFN induction by membrane fusion (134). It reflects a cGAS- and CDNs- independent STING activation.

Increased studies are showing that viruses inhibit the intracellular trafficking of STING. HSV-1 γ_1_34.5 protein perturbs STING trafficking from the ER to Golgi by interaction with STING on its N-terminus. STING is colocalizing with ER marker calreticulin in cells infected by wild type virus, while it forms puncta with GM130 (Golgi apparatus) in cells infected by γ_1_34.5 deletion viruses (135). It is still unknown how viral γ_1_34.5 protein inhibits STING trafficking. HCMV tegument protein UL82 is a negative regulator of the STING pathway by direct interaction with STING. It inhibits STING trafficking from the ER to perinuclear punctate structures by breaking the iRhom2-mediated assembly of the STING-TRAPβ translocation complex. STING fails to recruit TBK1 and IRF3 (101). Moreover, virulent African swine fever virus (ASFV) strain Armenia/07 attenuated STING-dependent IFN induction by re-localizing STING. ASFV is a complex, cytoplasmic dsDNA virus. STING colocalizes with clathrin adaptor protein AP1 outside from perinuclear structures in attenuated strain ASFV/NH/P68 but not in virulent strain Armenia/07 infected cells (136). With the increasing understanding of STING signaling transduction, more and more viral proteins would be found to manipulate the intracellular trafficking of the STING pathway.

## STING Trafficking, Autophagy and Recycling

Autophagy possesses important functions, including innate immune responses, and inflammation. Of note, dsDNA-induced STING trafficking involves autophagy (78). The autophagy-related gene ATG9A negatively regulates dsDNA-induced IFN expression by inhibiting the trafficking of STING. In ATG9A-deficient cells, translocation of STING to the perinuclear puncta and the assembly of STING-TBK1-IRF3 complexes are raised upon dsDNA stimulation (103). Moreover, knockdown of VPS34 (PI3KC3) inhibits STING trafficking and IFN induction by dsDNA stimulation. The Beclin-1-PI3KC3 (VPS34) core complex manipulates autophagy by generating PtdIns 3-phosphate-rich membranes, which are regarded as the platform for the recruitment of autophagy-related proteins and autophagosome maturation (137, 138). Beclin-1 interacts with cGAS and decreases STING induced IFN expression by repressing the enzymatic activity of cGAS (94). cGAS is required for dsDNA-induced Beclin-1 dependent autophagy (94). The cGAS–STING axis orchestrates ISGs and autophagy pathways to boost host immune responses to DNA viruses (93, 139–141). However, STING triggers autophagy independent of TBK1 activation and IFN induction. Above all, upon binding cGAMP, STING translocation to the Golgi is dependent on the COP-II complex and ARF GTPases. STING-coated ERGIC is the membrane source for LC3 lipidation, which initiates autophagosome maturation. cGAMP triggers LC3 lipidation by WIPI2 and ATG5 but independent of the ULK and VPS34-beclin-1 (78). LC3-positive membranes enfold dsDNA, bacteria, and viruses to form autophagosomes (142). Prabakaran and colleagues have found the interaction between STING and the selective autophagy receptor p62/SQSTM1, which attenuates cGAS-STING signaling. P62 is activated by TBK1-mediated phosphorylation. Phosphorylated p62 ubiquitinates STING and facilitates STING degradation by autophagy (143).

The translocation of STING plays a crucial role in the activation of downstream pathways. At the same time, dsDNA-induced autophagy is important for the removal of DNA and viruses in the cytoplasm. Upon cGAMP stimulation, STING induces autophagy but not IFN expression, indicating that autophagy induction is the original function of the cGAS-STING pathway (78, 144). Although the relationship between STING re-localization and autophagy has been established, the precision mechanism by which STING translocation is initiated remains unclear. Regarding the question of whether STING preferentially recruits IRF3 to perinuclear microsomes or via autophagosomes to activate the related immune response needs further exploration.

Moreover, STING translocates to the REs, and then to the p62-positive compartments/lysosomes after exiting from the Golgi apparatus (143). Chloroquine or BFA prevents the lysosomal degradation of STING and enhances STING-induced antiviral gene expression. The palmitoylation of STING is not required for its degradation because the palmitoylation-deficiency mutant (C88/91S) cannot prevent STING degradation. There are still controversies about the effect of the autophagic process in STING degradation (98).

## Post Translational Modifications and STING Regulation

Post-translational modification is important in the initiation, dynamic regulation, and silence of signal transduction pathways. It affects the pathway by regulation of protein localization, stabilization, and conformational changes (145). Examples of these regulations include ligand-dependent EGFR activation, Janus kinase (JAKs) regulated STAT signals, and ISG15-dependent regulation in TLR signals (146–148). The common types of PTMs are ubiquitination, phosphorylation (including serine/threonine phosphorylation and tyrosine phosphorylation), palmitoylation, glycation, lipidation, acetylation, methylation, and so on (149–151). It has been reported that ubiquitination, phosphorylation, and palmitoylation regulate the innate immune responses to dsDNA by STING. These modifications occur on all components in the pathway, including cGAS, STING, TBK1, and IRF3 (148).

Monoubiquitination and polyubiquitination regulate protein trafficking and degradation. K48-linked polyubiquitination is related to proteasomal degradation, while K63-linked polyubiquitination is related to signal transduction. Mostly, ubiquitin covalently binds to the lysine residue in substrate proteins through a multi-enzyme cascade, and the de-ubiquitination of proteins involves deubiquitinating enzymes (DUBs) (151, 152). However, it is clear now that polyubiquitin chains can also bind to substrates non-covalently. The E3-ligases TRIM32 and TRIM56 promote the recruitment of TBK1 by STING in response to the stimulus, by targeting STING for K63-linked ubiquitination at K150. Overexpression of these E3 ligases enhances IFNβ expression while knockout of either could abrogate STING-dependent responses. In a later study, researchers could not observe polyubiquitinations of STING in the presence of TRIM32 and TRIM56. The question about the precise coordination of TRIM32 and TRIM56 to STING in the process still remains to be elucidated (153–155).

Together with insulin-induced gene 1 (INSIG1), the autocrine motility factor receptor (AMFR) boost STING signaling by catalyzing a K27-linked polyubiquitination. Wang et al. reported that K27-linked di-ubiquitin chains bind the ubiquitin-like domain (ULD) of TBK1 directly (156). Four lysine residues of STING, K137, K150, K224, and K236, may involve in this process. However, it becomes controversial if the K27-linked polyubiquitination of STING is essential for TBK1 recruitment, since earlier studies are showing that TBK1 binds *Escherichia coli* derived recombinant STING fragments (84). RNF5 impairs STING signaling by modification of STING at K150 with K48-linked polyubiquitination, which promotes STING degradation. RNF26 catalyzes a K11-linked polyubiquitination at the same residue to antagonize RNF5-mediated STING degradation (157–159). The detailed regulations of TRIM32-, TRIM56-, RNF5-, and RNF26- dependent STING K150 polyubiquitination are worth exploring. Meanwhile, TRIM30α negatively regulates the STING pathway by the K48-linked ubiquitination of STING on K275. Knockdown and deficiency of TRIM30α enhance the production IFN-I and IL-6 upon dsDNA stimulation, and TRIM30α^–/–^ mice are more resistant to HSV-1 infection than wild type mice. Detailed studies show that TRIM30 interacts with STING through its SPRY domain (160, 161). Since TRIM30 could be induced by STING-NF-κB in response to dsDNA, it suggests that TRIM30 is a self-negative regulation component in STING signaling (161). It is worth noting that TRIM30α is absent in human (162). The E3-ligases TRIM29 inhibits the expression of STING and catalyzes the K48-linked ubiquitination of STING on K370. In the presence of cytoplasmic DNA, TRIM29 is highly expressed and impairs the expression of IFN-I. It is suggested that TRIM29^–/–^ mice are less susceptible to HSV-1 or adenovirus infection than wild type mice. TRIM29 plays a similar role as TRIM30 to inhibit innate immune responses (163). In addition, CD40 is reported to regulate the K48-linked ubiquitination of STING. The ubiquitin-ligase TRAFs are involved in the ubiquitination and stability of STING. Increased level of CD40 competes with STING to interact with TRAFs, reduces the degradation of STING, and promotes STING-mediated IFN-I responses (164). The mitochondrial E3 ubiquitin protein ligase 1 (MUL1) catalyzes K63-linked polyubiquitination of STING at K224, and deliver TBK1 to IRF3. It is found that the ubiquitination-deficient mutant STING K224R fails to translocate to perinuclear puncta in response to the stimulus, suggesting K63-linked polyubiquitination of STING at K224 is essential for STING trafficking (165). Interestingly, the MUL1- mediated STING ubiquitination is required in STING-IRF3 activation but not STING-NF-κB signals. It is noting that the dominant ubiquitination of STING on K236 and K338 are found in the same study (165).

As mentioned above, iRhom2 boosts gene induction by STING in responses to DNA viruses. It recruits the eukaryotic translation initiation factor 3, subunit 5 (EIF3S5) to STING, which removes K48-linked polyubiquitin of STING and inhibits STING degradation by the proteasome (106). USP13, a deubiquitinating enzyme, interacts with STING and catalyzed removal of K27 O- or K33 O-linked but not K27 R-linked polyubiquitin chains from STING. It impairs the recruitment of TBK1 by STING (166). USP13^*m/m*^ mice are more resistant to HSV-1 infection with a higher survival rate and a robuster IFN and cytokines in sera (166). Many viral proteins have already been found to de-ubiquitinate STING, which is discussed earlier in this review.

Palmitoylation is an important form of protein posttranslational lipid modification for regulating protein transport, stability, and cellular localization (167, 168). Palmitoylation of STING is found after trafficking to the Golgi apparatus, which is essential for the activation of STING (169, 170). The palmitoylation inhibitor 2-bromopalmitate (2-BP) impairs IFN induction via STING. The STING C88/91S mutant, which is deficient in palmitoylation, cannot induce ISGs expression in the presence of STING stimulus. It is demonstrated that STING is palmitoylated at the Golgi, and this PTM is essential for STING signaling (98, 169). Small molecules C-178 and its derivatives inhibit STING- mediated gene expression by antagonizing palmitoylation of STING with a covalent bond between C88/91 residue of STING and compound (171). This unique lipidation of protein may be maintaining the active STING oligomer on ERGIC or Golgi apparatus.

Protein phosphorylation is involved in almost all biological processes and is regulated by both kinases and phosphatases. Phosphorylation of STING at residue S366 by TBK1 promoted the recruitment and activation of IRF3. However, it is reported that phosphorylation of activated STING at S366 by ULK1 inhibits the activation of IRF3 at an earlier time (121). In both studies, S366A mutant that mimics unphosphorylated STING has a greatly reduced IFNβ expression in response to the stimulus, it is more convinced to conclude that phosphorylation of STING at S366 residue is a positive regulation. Besides, the residue S358 of STING is reported to carry through the phosphorylation process (172). Protein phosphatase Mg^2+^/Mn^2+^ dependent 1A (PPM1A) dephosphorylates STING at S358 and suppresses the formation of perinuclear puncta, which leads to reduced responses. The relationships between S358 and S366 phosphorylation are still unclear. Collectively, these studies reveal the positive effect of phosphorylation on STING activation (173). Currently, it is reported that the ribosomal protein S6 kinase 1 (S6K1) interacts with phosphorylated STING and TBK1 to form the transduction complex (174). It is a piece of the missed parts in the regulation of STING pathways, and partially explains the function of phosphorylation of STING in this signal.

Although tyrosine phosphorylation accounts for a small percentage of all protein phosphorylation modifications, it is critical in many processes. Tyrosine phosphorylation of STING has been identified in a preliminary experiment, in which STING (MPYS in this work) has been detected in immunoprecipitated samples by anti-pTyr antibodies (175). In the following years, less has been known for tyrosine phosphorylation of STING. In 2015, researchers found that Bruton’s tyrosine kinase (BTK) positively regulates STING-dependent signaling. BTK belongs to the Tec family of cytoplasmic tyrosine kinases. It is vital for B cell receptor signaling and lymphopoiesis (176). BTK interacts with STING and DDX41 and then phosphorylates DDX41. Y364 and Y414 of DDX41 are critical for DNA recognition and binding to STING. Y414 phosphorylation increases its affinity to STING by increasing the number of hydrogen bonds and salt bridges with STING. The finding indicates the interaction between DDX41 and the transmembrane region of STING by the tyrosine phosphorylation of DDX41 (177). Later, it is found that phosphorylation of Y245 in STING is important for STING activation. PTPN1 and PTPN2 dephosphorylated STING at Y245 and then facilitated STING degradation by 20S proteasome (178).

## Therapeutic Agents Targeting STING

Considering the nodal role of STING in the innate immune responses against abnormal DNA and viral invasion, it is tempting to harness this activity for therapy. The STING agonists and antagonists are immunotherapy drugs suitable for a variety of diseases. STING antagonists are supposed to cure autoimmune diseases, while STING agonists would be used in anti-tumor and antiviral therapies. We summarize the recent advances of STING agonists here.

STING agonists could activate innate and following adaptive immune responses for the treatment of many diseases, especially for cancers, and infectious diseases. Vascular disrupting agents DMXAA (also known as Vadimezan or ASA404) is the first STING agonist utilized in clinical trials, which directly interacts with mouse STING to activate TBK1-IRF3 and induces IFNs and cytokines. DMXAA reduces HBV DNA replication intermediates in the livers of HBV-injected mice. Unfortunately, DMXAA can only bind to mouse STING (179). It has extremely good efficacy in the mouse model, but the clinical trials failed in phase III (73). Meanwhile, immunotherapy based on STING agonists has always been considered to sweep the field of tumor immunotherapy (180–182). Researchers have discovered and designed a series of molecules to develop an effective activator of STING. To mimic the native agonist, nucleotidic agonist ADU-S100 (also called ML-RR-S2-CDA or MIW815) was designed and tested in clinical trials (180). Following this strategy, Merck, GlaxoSmithKline, and Bristol-Myers Squibb have patented different nucleotidic agonists of STING. Non-nucleotidic agonist diABZI was optimized from a small molecule lead compound amidobenzimidazole (ABZI). It binds to STING with an IC_50_ of 20 ± 0.8 nM and inhibits STING induced IFN-I expression in cells with an EC_50_ of 130 ± 40 nM (75). Until now, many other STING agonists or activators were reported, including IACS-8803, IACS-8779, and CL656 (183–185). Except for DMXAA, other STING agonists are developed for tumor immunotherapy. It is also believed that STING agonists might be used in antiviral therapies. One of the potential advantages of these new molecules is that they can be transported through blood (186). This new immunotherapy drug greatly enhanced the adaptive immune function. On one hand, the immune mechanism targeting STING provides new ideas for the entire anti-tumor and antiviral immunotherapy researches. On the other hand, the new STING agonists have also promoted the emergence and clinical application of new immune drugs.

## Concluding Remarks

A series of studies in the recent years demonstrated a critical role of STING signaling in the recognition of pathogenic DNA as well as endogenous DNA, and therefore in autoimmune diseases and tumor immunity. However, there remains a number of key questions unaddressed. For instance, the precise mechanism of regulation of the STING trafficking from the ER to the Golgi complex remains to be determined. In addition, STING has TBK1-independent and cGAS-independent functions. How these processes are regulated is not yet completely clear.

There is also substantial interest in identifying STING agonists and antagonists. DMXAA activates murine STING *in vitro* and *in vivo*, and CDNs activate human STING, potentially inhibiting metastatic tumors. It has shown that STING agonists may become another dark horse for immunotherapy. Given that direct IFN administration causes flu-like symptoms and other adverse effects, using CDNs or other small-molecules may reduce these side effects and lead to a more plausible therapy strategy.

## Author Contributions

All authors listed have made contributions to the manuscript and approved it for publication.

## Conflict of Interest

The authors declare that the research was conducted in the absence of any commercial or financial relationships that could be construed as a potential conflict of interest.
